# Effects of lung and airway epithelial maturation cocktail on the structure of lung bud organoids

**DOI:** 10.1186/s13287-018-0943-9

**Published:** 2018-07-11

**Authors:** Esmeralda Magro-Lopez, Charlotte Palmer, Joana Manso, Isabel Liste, Alberto Zambrano

**Affiliations:** Functional Unit for Research into Chronic Diseases, Institute of Health Carlos III, Ctra. Majadahonda-Pozuelo Km 2, 28220 Madrid, Spain

**Keywords:** Human embryonic stem cells, Lung bud organoid, Dexamethasone, Lung epithelial maturation medium

## Abstract

Organoids from human pluripotent stem cells are becoming suitable models for studies of organ development, drug screening, regenerative medicine, and disease modeling. Three-dimensional minilungs in Matrigel culture have recently been generated from human embryonic stem cells. These particular organoids, named lung bud organoids, showed branching airway and early alveolar structures resembling those present in lungs from the second trimester of human gestation. We show here that the treatment of such organoids with a lung and airway epithelial maturation cocktail containing dexamethasone drives lung bud organoids to the formation of paddle-racquet like structures. This strategy may help to increase the versatility of lung organoids and to generate structures more advanced than the original branching texture.

## Main text

The respiratory system originates from buds arising from the anterior foregut endoderm (AFE) through well-defined stages of differentiation named the embryonic, pseudoglandular, canalicular, saccular, and alveolar stages. The generation of lung organoids should emulate this natural sequence of differentiation as far as possible. A number of authors have reported the generation of human lung organoids [1–3]. Dye et al. have shown the generation of small structures expressing markers of lung and airway cells, but neither branching morphogenesis nor proximodistal specification was observed [2, 3]. Snoeck’s group, however, recently described the generation of three-dimensional structures from human embryonic stem cells (hESCs) that were spatially organized in a similar way to developing lung buds in vivo; they called these lung bud organoids (LBOs). Briefly, their strategy was the following: AFE was generated from definitive endoderm (DE) in a two-dimensional sequential development as previously described [4, 5]. After that, adherent structures (during ventralization of AFE between days 6 and 8) were expanded in suspension as clumps of cells in the presence of bone morphogenetic protein (BMP) 4, fibroblast growth factor (FGF) 10, keratinocyte growth factor (KGF), the GSK3β antagonist CHIR99201, and retinoic acid (ventralization/branching medium). These LBOs structures were grown until day 25 and were then plated in Matrigel. After this, LBOs progressively underwent extensive outward branching reminiscent of saccules formed during the saccular stage of lung development and showed early signs of alveologenesis [1]. LBOs in Matrigel contained alveolar functional type II (ATII) epithelial cells with abundant lamellar bodies.

**Fig. 1:**
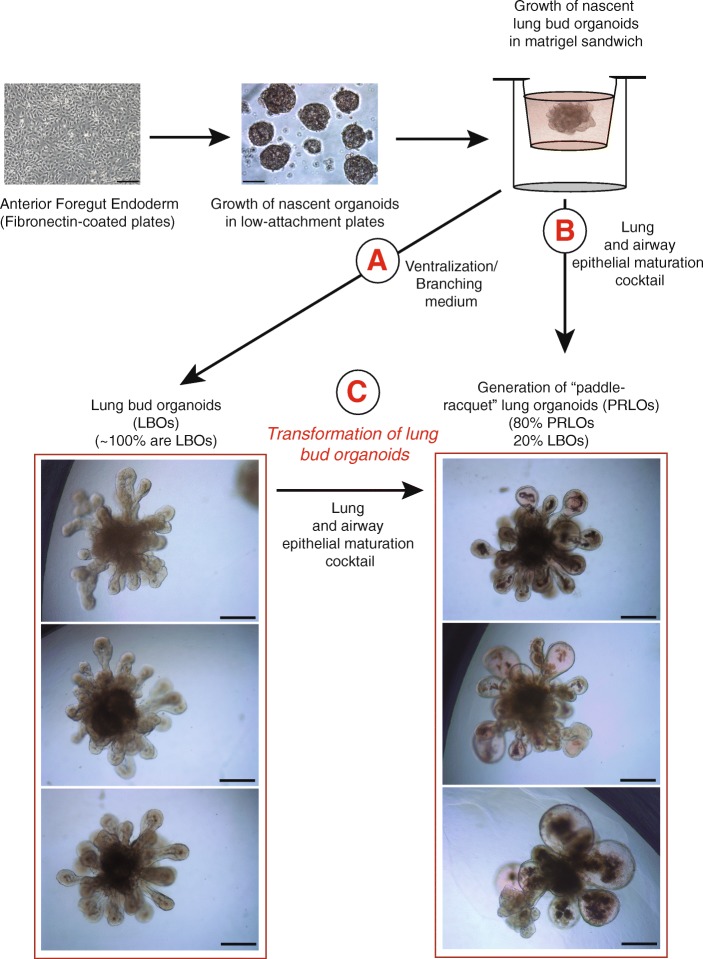
Strategy followed to generate human lung organoids. The essential sequential steps of hESC differentiation to lung bud organoids (LBOs) or paddle-racquet lung organoids (PRLOs) is shown. A, B, and C are the three paths followed (described in the text and in the Materials and methods section). Scale bars = 200 μm

**Fig. 2:**
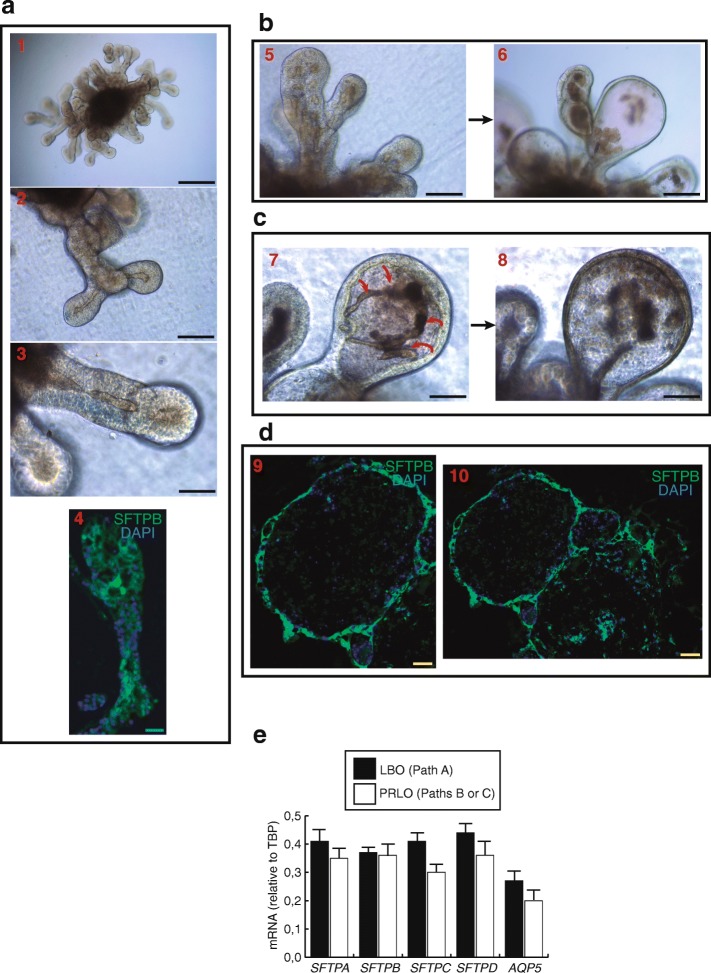
Morphological features of lung bud organoids (LBOs) and paddle-racquet lung organoids (PRLOs), and expression of alveolar cells markers. **a** Representative micrographs of LBOs and PRLOs. Pictures 1–3 are representative micrographs of LBOs at day 38 showing the typical finger-shaped extensions. Scale bars = 200 μm (pictures 1,2) and 100 μm (picture 3). Picture 4 shows the expression of surfactant protein B (SFTPB) by immunofluorescence. Scale bar = 50 μm. **b** Pictures 5 and 6 show the effect of the treatment with the lung and airway epithelial maturation cocktail (path “C”, see text); Scale bars = 200 μm. **c** Pictures 7 and 8 are representative micrographs showing the detachment of material from the inner surface of the growing PRLO. Scale bars = 100 μm. **d**. Pictures 9 and 10 are representative immunofluorescence micrographs showing the expression of SFTPB at the surface of PRLOs. Scale bars = 50 μm. **e** Expression of surfactant genes (ATII cell markers) and *AQP5* (ATI cell marker) by qRT-PCR

We have recently reported the generation of two-dimensional minilungs from hESCs following the strategy delineated by Huang et al. in previous reports [4, 5]. We included some modifications regarding the cellular densities in some replatings, the final assembly of the minilungs on glass chambers, and the addition of primary myofibroblasts in order to enrich the generated two-dimensional structures [6]. The generation of airway and lung epithelial cells implicated the generation of NKX2.1^+^/FOXA2^+^ cells that corresponded to the lung field (progenitors) of AFE, their expansion until day 26, and their terminal differentiation in the presence of a lung and airway maturation cocktail containing CHIR99021, FGF10, KGF, isobutylmethylxanthine (IBMX), 8-bromo-cAMP, and dexamethasone. IBMX, 8-bromo-cAMP, and dexamethasone, are factors that induce alveolar maturation in fetal mouse lung explants and increase surfactant protein expression in mouse ESC-derived lung progenitors [7, 8]. This in-vitro differentiation protocol yielded at least six types of lung and airways cells but was biased toward distal cells. To generate three-dimensional structures, we employed the hESC line AND-1 [6] and followed the LBO formation strategy described by Chen et al. [1]. Some modifications in the protocol were included (see Materials and methods). Essentially, after AFE formation, cells were briefly trypsinized into small 3–10 cell clumps. These clumps were plated onto low-attachment plates in ventralization/branching medium. These three-dimensional clumps (nascent lung bud organoids) were incubated and fed every other day for approximately 20–25 days. After that, these nascent organoids were embedded into a Matrigel sandwich assembled on MW12 inserts (or transwells). Growing branching structures were easily visualized under the microscope after 1 or 2 weeks of treatment. However, in contrast to the strategy of Chen et al. which is based on the ventralization/branching medium, we also grew the nascent LBOs in the presence of a lung and alveolar epithelial maturation cocktail containing CHIR99021, FGF10, KGF, IBMX, and 8-bromo-cAMP. The aim of this was to test whether factors necessary for the maturation of lung epithelial cells could give rise to the generation of structures more advanced than the saccular stage. Figure 1 shows the overall strategy followed. After the early generation of nascent LBOs, we either grew the organoids in the presence of the ventralization/branching medium (path “A”; *n* = 56) or in the presence of the lung and alveolar epithelial maturation cocktail until approximately 65–70 days (path “B”; *n* = 20). In addition, a number of LBOs were also treated with the lung and alveolar epithelial maturation cocktail at day 70 for approximately 20 days (path “C”; *n* = 20). The incubation with ventralization/branching medium (path “A”) consistently yielded LBO structures matching those described by Chen et al. [1] (*n* = 56; *P* < 0,0001). However, the incubation with the lung and alveolar epithelial maturation cocktail (paths “B” or “C”) produced approximately 20% LBO organoids and 80% organoids that we named “paddle-racquet” lung organoids (PRLOs) (*n* = 40; *P* < 0,001). These organoids consisted of rounded expansions of the lung buds sometimes showing partitioned expansions as shown in Fig. 1 or the presence of large structures resembling alveolospheres. A striking feature of these PRLOs is the presence of a dense material in the center of the rounded expansion. This material probably has its origin in the columnar epithelium of the original LBOs, as some material seems to detach from the inner surface and fall into the lumen of the PRLOs (Fig. 1). Figure 2a shows these observations in more detail. Pictures 1–3 show the finger-shaped extensions occurring during the development of LBOs. The expression of mature surfactant protein (SFTP) B was analyzed by indirect immunofluorescence of typical LBOs. As shown in Fig. 2a (picture 4) the expression of SFTPB was abundant and localized predominantly to the tips of the extensions as previously described [1]. Figure 2b (pictures 5 and 6) shows the result of the incubation of LBOs with the lung and alveolar epithelial maturation cocktail (path “C”) for 20 days. A dense material, probably coming from the original columns of LBOs, is present in the lumen of PRLOs. Picture 7 (Fig. 2c) shows a strip of columnar material that is detaching from the inner surface. As a result of this, the PRLOs are composed of thin surfaces larger than those of the LBOs. The analysis of these PRLOs by immunofluorescence showed the presence of abundant SFTPB expression in cells forming the rounded extensions of the PRLOs (Fig. 2d). As it appears from the immunofluorescence result, the structural changes observed did not seem to be due to a net loss of ATII cells but were rather due to overall structure remodeling. According to this, the expression levels of surfactant genes and of an ATI cell marker (*AQP5*) observed by quantitative real-time polymerase chain reaction (qRT-PCR) were very similar, showing no significant differences (Fig. 2e). Taken together, these results show that factors inducing lung and alveolar epithelial maturation may alter the structure of LBOs favoring the formation of nascent alveolar structures that could anticipate the entry into the alveolar stage of development. Our strategy may also help to increase the versatility of lung organoids and to generate structures to model diseases such as congenital surfactant deficiency syndromes [9].

## Materials and methods

### Maintenance of hESCs

AND-1, a human embryonic stem cell line, was obtained from the “Biobanco de células madre de Granada” (ISCIII, Spain) at passages 27–40. Mouse embryonic fibroblasts (MEFs) were obtained at 13.5 days postcoitum from C57BL/6 mice as described previously [10]. This line was karyotyped and verified for mycoplasma contamination. MEFs were mitotically inactivated by overnight treatment with 2 μg/mL mitomycin C (cat. no. M4287; Sigma-Aldrich) and plated at a density of approximately 16,000 cells/cm^2^. AND-1 cells were cultured on MEFs under standard conditions (http://stembook.org)). The maintenance medium was composed of knockout Dulbecco’s modified Eagle’s medium (KO-DMEM; cat. no. 10829–018 Gibco; Life Technologies), 20% KO serum replacement (cat. no. 10828–010 Gibco; Life Technologies), 0.1 mM β-mercaptoethanol (cat. no. 21985–023 Gibco; Life Technologies), 2 mM Glutamax (cat. no. 35050–061, Gibco; Life Technologies), nonessential amino acids (cat. no. 11140–050 Gibco; Life Technologies), and primocin (cat. no. 12I05-MM, InvivoGen). The medium was filtered using a 0.22-μm pore filter system (cat. no. 431097, Corning), and 10 ng/mL recombinant human basic fibroblast growth factor (hbFGF; cat. no. PHG6015, Invitrogen) and 10 μM Y-27632 (cat. no. 1254, Tocris R&D Systems) were added before use. Medium was changed daily and cells were passaged either by enzymatic (the collagenase IV method) (collagenase IV; cat. no. 11140–050, Gibco; Life Technologies) or mechanical procedures (http://stembook.org). Cells were maintained in an undifferentiated state in a 5% CO_2_/air environment. The differentiation process was carried out under normoxic conditions unless otherwise indicated.

### Primitive streak formation and induction of DE

Induction of endoderm was carried out as previously described [6]. Briefly, primitive streak formation and endoderm induction were performed in serum-free differentiation (SFD) medium. SFD medium was composed of a mix of IMDM:F12 (3:1) media (cat. nos. B12-722F and 10–080 CVR, Corning), supplemented with N2 (cat. no. 17502–048, Gibco; Life Technologies), B27 (cat. no. 17504–044, Gibco; Life Technologies), 2 mM Glutamax (cat. no. 35050–061 Gibco; Life Technologies), 1% penicillin-streptomycin (DE17-602E, Lonza), and 0.05% bovine serum albumin (BSA; cat. no. A7906, Sigma-Aldrich). The medium was filtered using a 0.22-μm pore filter system (cat. no. 431097, Corning), and 50 μg/mL ascorbic acid (cat. no. A4554, Sigma-Aldrich) and 0.04 μL/mL monothioglycerol (stock >97%, cat. no. M6145, Sigma-Aldrich) were added before use. MEFs were depleted by passaging AND-1 onto Matrigel-coated (cat. no. 354230, Life Technologies) plates. Embryoid bodies (EBs) were formed in low-attachment six-well plates (cat. no. 3471, Corning) and maintained in SFD medium in a 5% CO_2_/5% O_2_/95% N_2_ environment (Galaxy 48R incubator; New Brunswick). For primitive streak formation, 10 μM Y-27632, 10 ng/mL Wnt3a (cat. no. 5036-WN, R&D Systems) and 3 ng/mL human BMP4 (cat. no. 314-BP, R&D Systems) was used. EBs were collected, resuspended carefully in endoderm induction medium containing 10 μM Y-27632, 0.5 ng/mL human BMP4, 2.5 ng/mL hbFGF, and 100 ng/mL human activin (cat. no. 338-AC, R&D Systems). Cells were fed after 36–48 h depending on cell density by removing half the old medium and adding half fresh medium.

### Induction of AFE

AFE was induced as previously described [6]. Briefly, EBs were dissociated into single cells with trypsin. Dissociated cells were transferred to a conical tube containing stop medium to neutralize the trypsin. Cells were centrifuged for 5 min at 850 rpm, washed carefully twice with SFD medium and counted. For AFE induction, 25,000–30,000 cells/cm^2^ were plated on fibronectin-coated (F0895, Sigma-Aldrich) 12-well tissue culture plates in AFE induction medium 1 (SFD medium supplemented with 10 mM SB-431542 (cat. no. 1614, Tocris) and 100 ng/mL of NOGGIN (cat. no. 6057, R&D Systems)). After 24 h of incubation, the medium was aspirated and AFE induction medium 2 (SFD medium supplemented with 1 μM IWP2 (cat. no. 3533, Tocris) and 10 μM of SB-431542) was added to the cultures. This process was carried out under normoxic conditions.

### Formation of lung bud organoids

After AFE formation, cells were briefly trypsinized into small 3–10 cell clumps and the reaction was halted with stop medium (IMDM medium (BE12-722F) supplemented with 50% fetal bovine serum (FBS; F7524, Sigma-Aldrich), 2 mM Glutamax, 1% penicillin-streptomycin). Cells were then centrifuged for 5 min at 850 rpm and washed carefully twice with an excess of SFD medium. The clumps were plated onto low-attachment six-well plates (cat. no. 3471, Corning) in branching medium (SFD medium containing 3 μM CHIR99021, 10 ng/mL FGF10, 10 ng/mL KGF, 10 ng/mL BMP4, 50 nM *all-trans* retinoic acid). These three-dimensional clumps (nascent lung bud organoids) were incubated and fed every other day for approximately 20–25 days. After that, these nascent organoids were embedded into a Matrigel sandwich assembled on MW12 inserts (or transwells). Briefly, 100 μL Matrigel was loaded on the transwell insert and allowed to gel. Then nascent organoids were picked up with a wide mouth plastic Pasteur pipette, divided into MW24 wells containing 50% Matrigel diluted in branching media, and immediately transferred onto the first layer of Matrigel. After solidification of this intermediate layer containing the nascent organoids, 100 μL Matrigel was added on top. Finally, each sandwich containing various organoids was incubated with 200 μL branching media inside the transwell, and 500 μL around it. Medium inside the transwell was changed every 2–3 days. Around the transwells, the medium was added on a regular basis to maintain the bottom of the insert in continuous contact with the medium. Growing branching structures were easily visualized under the microscope after 1 or 2 weeks. These lung bud organoids or three-dimensional minilungs were treated or not with the lung and alveolar epithelial maturation cocktail (SFD medium containing 3 μM CHIR99021, 10 ng/mL FGF10, 10 ng/mL KGF, 0.1 mM IBMX, 0.1 mM 8-bromo-cAMP, and 60 nM dexamethasone) at the indicated times.

### Indirect immunofluorescence of lung bud organoids

Organoids were picked up from the MW12 inserts, transferred into a well of a MW12 and fixed with 4% paraformaldehyde (PFA) for 15 min at room temperature. After that, the organoids were washed three times with phosphate-buffered saline (PBS) and incubated overnight at 4 °C with 30% sucrose. The sucrose was then exchanged for a solution of 7.5% gelatin/15% sucrose and incubated for 15 min at 37 °C. Organoids were then transferred to cryomolds containing solidified 7.5% gelatin/15% sucrose and were progressively embedded in various layers of solidified 7.5% gelatin/15% sucrose. These preparations were cut into 10-μm sections in a Leica CM3050 cryostat. Organoid sections were analyzed by indirect immunofluorescence. Briefly, the sections were washed with PBS and permeabilized with PBS/1% BSA/0.25% Triton X-100 for 5 min at room temperature. After that, the sections were washed and blocked for 30 min at room temperature with blocking solution (PBS/1% BSA). The sections were incubated for 2 h with an antibody against surfactant protein B (cat. no. sc-133143, Santa Cruz Biotech). Preparations were then washed with washing solution and incubated with a secondary antibody conjugated with Alexa fluor dye (488) from Life Technologies (A-11029) for 1 h at room temperature. Nuclei were counterstained with DAPI and samples were mounted with ProLong Diamond (P36961; Life Technologies). Cell images were captured with a fluorescence microscopy (Zeiss Axio) equipped with a camera (Axiocam MRm) and AxioVision software.

### qRT-PCR

RNA extraction and RT reactions were performed with Trizol reagent (cat. no. 15596026; Ambion) and the high-capacity cDNA kit (cat. no. 4387406; Applied Biosystems) following the manufacturer’s instructions. Real-time PCR was performed using the powerUp SYBR Green mix (cat. no. A25742) and the Quantstudio-3 system from Applied Biosystems. The relative amounts of the amplification products were calculated by the ΔΔCt method. The genes analyzed, and the sequences of the oligonucleotides employed in this study, are shown in Table 1.

**Table 1:** The genes analyzed, and the sequences of the oligonucleotides employed in this study

Gene	Oligonucleotides sequences
*SRFTPA*	5’-GTGCGAAGTGAAGGACGTTTGTGT 5’-TTTGAGACCATCTCTCCCGTCCC
*SRFTPB*	5’-TCTGAGTGCCACCTCTGCATGT 5’-TGGAGCATTGCCTGTGGTATGG
*SRFTPC*	5’-CCTTCTTATCGTGGTGGTGGTGGT 5’-TCTCCGTGTGTTTCTGGCTCATGT
*SRFTPD*	5’-TGACTGATTCCAAGACAGAGGGCA 5’-TCCACAAGCCCTGTCATTCCACTT
*AQP5*	5′- GCCATCCTTTACTTCTACCTGCTC 5′- GCTCATACGTGCCTTTGATGATGG

### Statistical analysis

Statistical significance of data was determined by the analysis of variance followed by the Newman–Keuls or Bonferroni post-hoc tests for the experiments with three experimental groups. *P* < 0.05 was considered significant. Significance of the analysis of variance test is indicated in the text. Statistics were calculated with GraphPad Prism 7 software. Experiments were repeated at least two times.

## Abbreviations

AFE: Anterior foregut endoderm
ATII: Alveolar type II cell
BMP: Bone morphogenetic protein
BSA: Bovine serum albumin
DE: Definitive endoderm
EB: Embryoid body
FBS: Fetal bovine serum
FGF: Fibroblast growth factor
hbFGF: Human basic fibroblast growth factor
hESC: Human embryonic stem cell
IBMX: Isobutylmethylxanthine
KGF: Keratinocyte growth factor
LBO: Lung bud organoid
MEF: Mouse embryonic fibroblast
PBS: Phosphate-buffered saline
PRLO: Paddle-racquet lung organoid
qRT-PCR: Quantitative real-time polymerase chain reaction
SFD: Serum-free differentiation
SFTP: Surfactant protein
